# Supplementary material for: Cell Line-Dependent Effects of Spheroid Formation Method on Drug Response in Melanoma Models
Source: Cells. 2026 Jun 11;15(12):1069. doi: 10.3390/cells15121069 (PMC13297100; doi:10.3390/cells15121069)
Supplement: Supplementary file 1 [file cells-15-01069-s001.zip › cells-4341203-supplementary.pdf]

Supplementary material

Cell Line-Dependent Effects of Spheroid Formation Method on Drug Response in Melanoma Models

Akvilė Žilytė [1] and Vilma Petrikaitė [1,2*]

[1] Laboratory of Drug Targets Histopathology, Institute of Cardiology, Lithuanian University of Health Sciences, Sukilėlių Av. 13, LT-50162 Kaunas, Lithuania; akvile.zilyte@stud.lsmu.lt
[2] Institute of Biotechnology, Life Sciences Center, Vilnius University, Saulėtekio 7, LT-10257 Vilnius, Lithuania
* Correspondence: vilma.petrikaite@lsmu.lt, vilma.petrikaite@bti.vu.lt; Tel.: +370-68629383

Procedure S1. Evaluation of compound cytotoxicity in a cell monolayer

The effect of DOX and 5-FU on cell viability was established using the 3-(4,5-dimethylthiazol-2-yl)-2,5-diphenyltetrazolium bromide (MTT; Sigma-Aldrich Co., St Louis, MO, USA) assay. The cells were cultured, and the cell suspension was prepared as described in the 2.1 section. Then the cells were seeded in triplicate in 96-well TC-treated flat-bottom plates (4 × 10^3 cells/well). The compounds were serially diluted and added to the cells after 24 hours of incubation. After 72 h, the MTT reagent was added, and the formed formazan crystals were dissolved in 2-propanol (Sigma-Aldrich Co., St. Louis, MO, USA). The absorbance was determined with a multidetection microplate reader at 570 and 630 nm. The Hill equation was used to calculate the EC_{50} value, or the concentration of a compound that results in a 50% reduction in the metabolic activity of cells.

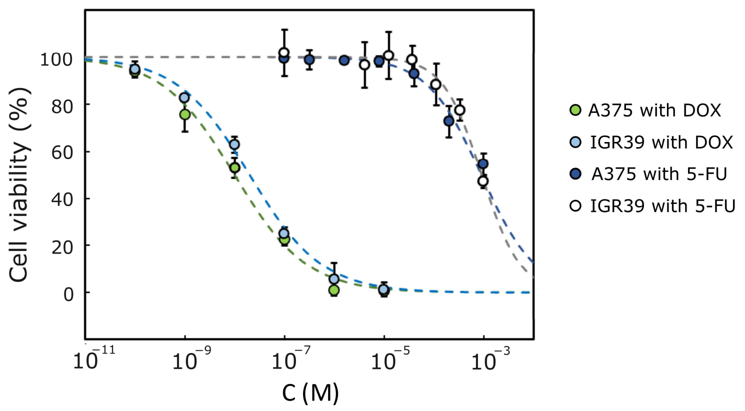

Figure S1. Representative dose-response curves for anticancer compounds DOX and 5-FU in melanoma cell lines A375 and IGR39, after cell incubation with tested compounds for 72 hours. The dose-effect correlation has been calculated using the Hill equation. Data points are experimental values (averages of three repeats), while the lines are a fit of the standard inhibition model.
